# Contact Tracing during Coronavirus Disease Outbreak, South Korea, 2020

**DOI:** 10.3201/eid2610.201315

**Published:** 2020-10

**Authors:** Young Joon Park, Young June Choe, Ok Park, Shin Young Park, Young-Man Kim, Jieun Kim, Sanghui Kweon, Yeonhee Woo, Jin Gwack, Seong Sun Kim, Jin Lee, Junghee Hyun, Boyeong Ryu, Yoon Suk Jang, Hwami Kim, Seung Hwan Shin, Seonju Yi, Sangeun Lee, Hee Kyoung Kim, Hyeyoung Lee, Yeowon Jin, Eunmi Park, Seung Woo Choi, Miyoung Kim, Jeongsuk Song, Si Won Choi, Dongwook Kim, Byoung-Hak Jeon, Hyosoon Yoo, Eun Kyeong Jeong, on behalf of the COVID-19 National Emergency Response Center, Epidemiology and Case Management Team

**Affiliations:** Korea Centers for Disease Control and Prevention, Cheongju, South Korea (Y.J. Park, O. Park, S.Y. Park, Y.-M. Kim, J. Kim, S. Kweon, Y. Woo, J. Gwack, S.S. Kim, J. Lee, J. Hyun, B. Ryu, Y.S. Jang, H. Kim, S.H. Shin, S. Yi, S. Lee, H.K. Kim, H. Lee, Y. Jin, E. Park, S.W. Choi, M. Kim, J. Song, S.W. Choi, D. Kim, B.-H. Jeon, H. Yoo, E.K. Jeong);; Hallym University College of Medicine, Chuncheon, South Korea (Y.J. Choe)

**Keywords:** coronavirus disease, 2019 novel coronavirus disease, COVID-19, SARS-CoV-2, severe acute respiratory syndrome coronavirus 2, respiratory diseases, zoonoses, viruses, pneumonia, South Korea, contact tracing

## Abstract

We analyzed reports for 59,073 contacts of 5,706 coronavirus disease (COVID-19) index patients reported in South Korea during January 20–March 27, 2020. Of 10,592 household contacts, 11.8% had COVID-19. Of 48,481 nonhousehold contacts, 1.9% had COVID-19. Use of personal protective measures and social distancing reduces the likelihood of transmission.

Effective contact tracing is critical to controlling the spread of coronavirus disease (COVID-19) (*1*). South Korea adopted a rigorous contact-tracing program comprising traditional shoe-leather epidemiology and new methods to track contacts by linking large databases (global positioning system, credit card transactions, and closed-circuit television). We describe a nationwide COVID-19 contact tracing program in South Korea to guide evidence-based policy to mitigate the pandemic (*2*).

## The Study

South Korea’s public health system comprises a national-level governance (Korea Centers for Disease Control and Prevention), 17 regional governments, and 254 local public health centers. The first case of COVID-19 was identified on January 20, 2020; by May 13, a total of 10,962 cases had been reported. All reported COVID-19 patients were tested using reverse transcription PCR, and case information was sent to Korea Centers for Disease Control and Prevention.

We defined an index case as the first identified laboratory-confirmed case or the first documented case in an epidemiologic investigation within a cluster. Contacts in high-risk groups (household contacts of COVID-19 patients, healthcare personnel) were routinely tested; in non–high-risk groups, only symptomatic persons were tested. Non–high-risk asymptomatic contacts had to self-quarantine for 14 days and were placed under twice-daily active surveillance by public health workers. We defined a household contact as a person who lived in the household of a COVID-19 patient and a nonhousehold contact as a person who did not reside in the same household as a confirmed COVID-19 patient. All index patients were eligible for inclusion in this analysis if we identified >1 contact. We defined a detected case as a contact with symptom onset after that of a confirmed COVID-19 index patient.

We grouped index patients by age: 0–9, 10–19, 20–29, 30–39, 40–49, 50–59, 60–69, 70–79, and >80 years. Because we could not determine direction of transmission, we calculated the proportion of detected cases by the equation [number of detected cases/number of contacts traced] × 100, excluding the index patient; we also calculated 95% CIs. We compared the difference in detected cases between household and nonhousehold contacts across the stratified age groups.

We conducted statistical analyses using RStudio (https://rstudio.com). We conducted this study as a legally mandated public health investigation under the authority of the Korean Infectious Diseases Control and Prevention Act (nos. 12444 and 13392).

We monitored 59,073 contacts of 5,706 COVID-19 index patients for an average of 9.9 (range 8.2–12.5) days after severe acute respiratory syndrome coronavirus 2 (SARS-CoV-2) infection was detected (Table 1). Of 10,592 household contacts, index patients of 3,417 (32.3%) were 20–29 years of age, followed by those 50–59 (19.3%) and 40–49 (16.5%) years of age (Table 2). A total of 11.8% (95% CI 11.2%–12.4%) of household contacts of index patients had COVID-19; in households with an index patient 10–19 years of age, 18.6% (95% CI 14.0%–24.0%) of contacts had COVID-19. For 48,481 nonhousehold contacts, the detection rate was 1.9% (95% CI 1.8%–2.0%) (Table 2). With index patients 30–39 years of age as reference, detection of COVID-19 contacts was significantly higher for index patients >40 years of age in nonhousehold settings. For most age groups, COVID-19 was detected in significantly more household than nonhousehold contacts (Table 2).

**Table 1:** Contacts traced by age group of index coronavirus disease patients, South Korea, January 20–March 27, 2020

Index patient age, y	No. (%) index patients	No. (%) contacts traced	No. contacts traced/index patient	Average time contacts monitored, d
0–9	29 (0.5)	237 (0.4)	8.2	12.5
10–19	124 (2.2)	457 (0.8)	3.7	9.0
20–29	1,695 (29.7)	15,810 (26.8)	9.3	9.8
30–39	668 (11.7)	8,636 (14.6)	12.9	11.1
40–49	807 (14.1)	9,709 (16.4)	12.0	11.0
50–59	1,107 (19.4)	11,353 (19.2)	10.3	9.6
60–69	736 (12.9)	8,490 (14.4)	11.5	8.2
70–79	338 (5.9)	2,389 (4.0)	7.1	8.5
>80	202 (3.5)	1,992 (3.4)	9.9	9.4
Total	5,706	59,073	10.4	9.9

**Table 2:** Rates of coronavirus disease among household and nonhousehold contacts, South Korea, January 20–March 27, 2020

Index patient age, y	Household			Nonhousehold	
	No. contacts positive/no. contacts traced	% Positive (95% CI)		No. contact positive/no. contacts traced	% Positive (95% CI)
0–9	3/57	5.3 (1.3–13.7)		2/180	1.1 (0.2–3.6)
10–19	43/231	18.6 (14.0–24.0)		2/226	0.9 (0.1–2.9)
20–29	240/3,417	7.0 (6.2–7.9)		138/12,393	1.1 (0.9–1.3)
30–39	143/1,229	11.6 (9.9–13.5)		70/7,407	0.9 (0.7–1.2)
40–49	206/1,749	11.8 (10.3–13.4)		161/7,960	2.0 (1.7–2.3)
50–59	300/2,045	14.7 (13.2–16.3)		166/9,308	1.8 (1.5–2.1)
60–69	177/1,039	17.0 (14.8–19.4)		215/7,451	2.9 (2.5–3.3)
70–79	86/477	18.0 (14.8–21.7)		92/1,912	4.8 (3.9–5.8)
≥80	50/348	14.4 (11.0–18.4)		75/1,644	4.6 (3.6–5.7)
Total	1,248/10,592	11.8 (11.2–12.4)		921/48,481	1.9 (1.8–2.0)

## Conclusions

We detected COVID-19 in 11.8% of household contacts; rates were higher for contacts of children than adults. These risks largely reflected transmission in the middle of mitigation and therefore might characterize transmission dynamics during school closure (*3*). Higher household than nonhousehold detection might partly reflect transmission during social distancing, when family members largely stayed home except to perform essential tasks, possibly creating spread within the household. Clarifying the dynamics of SARS-CoV-2 transmission will help in determining control strategies at the individual and population levels. Studies have increasingly examined transmission within households. Earlier studies on the infection rate for symptomatic household contacts in the United States reported 10.5% (95% CI 2.9%–31.4%), significantly higher than for nonhousehold contacts (*4*). Recent reports on COVID-19 transmission have estimated higher secondary attack rates among household than nonhousehold contacts. Compiled reports from China, France, and Hong Kong estimated the secondary attack rates for close contacts to be 35% (95% CI 27%–44%) (*5*). The difference in attack rates for household contacts in different parts of the world may reflect variation in households and country-specific strategies on COVID-19 containment and mitigation. Given the high infection rate within families, personal protective measures should be used at home to reduce the risk for transmission (*6*). If feasible, cohort isolation outside of hospitals, such as in a Community Treatment Center, might be a viable option for managing household transmission (*7*).

We also found the highest COVID-19 rate (18.6% [95% CI 14.0%–24.0%]) for household contacts of school-aged children and the lowest (5.3% [95% CI 1.3%–13.7%]) for household contacts of children 0–9 years in the middle of school closure. Despite closure of their schools, these children might have interacted with each other, although we do not have data to support that hypothesis. A contact survey in Wuhan and Shanghai, China, showed that school closure and social distancing significantly reduced the rate of COVID-19 among contacts of school-aged children (*8*). In the case of seasonal influenza epidemics, the highest secondary attack rate occurs among young children (*9*). Children who attend day care or school also are at high risk for transmitting respiratory viruses to household members (*10*). The low detection rate for household contacts of preschool-aged children in South Korea might be attributable to social distancing during these periods. Yet, a recent report from Shenzhen, China, showed that the proportion of infected children increased during the outbreak from 2% to 13%, suggesting the importance of school closure (*11*). Further evidence, including serologic studies, is needed to evaluate the public health benefit of school closure as part of mitigation strategies.

Our observation has several limitations. First, the number of cases might have been underestimated because all asymptomatic patients might not have been identified. In addition, detected cases could have resulted from exposure outside the household. Second, given the different thresholds for testing policy between households and nonhousehold contacts, we cannot assess the true difference in transmissibility between households and nonhouseholds. Comparing symptomatic COVID-19 patients of both groups would be more accurate. Despite these limitations, the sample size was large and representative of most COVID-19 patients early during the outbreak in South Korea. Our large-scale investigation showed that pattern of transmission was similar to those of other respiratory viruses (*12*). Although the detection rate for contacts of preschool-aged children was lower, young children may show higher attack rates when the school closure ends, contributing to community transmission of COVID-19.

The role of household transmission of SARS-CoV-2 amid reopening of schools and loosening of social distancing underscores the need for a time-sensitive epidemiologic study to guide public health policy. Contact tracing is especially important in light of upcoming future SARS-CoV-2 waves, for which social distancing and personal hygiene will remain the most viable options for prevention. Understanding the role of hygiene and infection control measures is critical to reducing household spread, and the role of masking within the home, especially if any family members are at high risk, needs to be studied.

We showed that household transmission of SARS-CoV-2 was high if the index patient was 10–19 years of age. In the current mitigation strategy that includes physical distancing, optimizing the likelihood of reducing individual, family, and community disease is important. Implementation of public health recommendations, including hand and respiratory hygiene, should be encouraged to reduce transmission of SARS-CoV-2 within affected households.

## References

[1] World Health Organization. Contact tracing in the context of COVID-19 [cited 2020 May 15]. https://www.who.int/publications/i/item/contact-tracing-in-the-context-of-covid-19

[2] COVID-19 National Emergency Response Center. Epidemiology & Case Management Team, Korea Centers for Disease Control & Prevention. Contact transmission of COVID-19 in South Korea: novel investigation techniques for tracing contacts. Osong Public Health Res Perspect. 2020;11:60–3. 10.24171/j.phrp.2020.11.1.09 32149043 PMC7045882

[3] Choe YJ, Choi EH. Are we ready for coronavirus disease 2019 arriving at schools? J Korean Med Sci. 2020;35:e127. 10.3346/jkms.2020.35.e127 32193906 PMC7086087

[4] Burke RM, Midgley CM, Dratch A, Fenstersheib M, Haupt T, Holshue M, et al. Active monitoring of persons exposed to patients with confirmed COVID-19—United States, January–February 2020. MMWR Morb Mortal Wkly Rep. 2020;69:245–6. 10.15585/mmwr.mm6909e1 32134909 PMC7367094

[5] Liu Y, Eggo RM, Kucharski AJ. Secondary attack rate and superspreading events for SARS-CoV-2. Lancet. 2020;395:e47. 10.1016/S0140-6736(20)30462-1 32113505 PMC7158947

[6] Jefferson T, Del Mar C, Dooley L, Ferroni E, Al-Ansary LA, Bawazeer GA, et al. Physical interventions to interrupt or reduce the spread of respiratory viruses: systematic review. BMJ. 2009;339(sep21 1):b3675. 10.1136/bmj.b3675 PMC2749164 19773323

[7] Park PG, Kim CH, Heo Y, Kim TS, Park CW, Kim CH. Out-of-hospital cohort treatment of coronavirus disease 2019 patients with mild symptoms in Korea: an experience from a single community treatment center. J Korean Med Sci. 2020;35:e140. 10.3346/jkms.2020.35.e140 32242347 PMC7131899

[8] Zhang J, Litvinova M, Liang Y, Wang Y, Wang W, Zhao S, et al. Changes in contact patterns shape the dynamics of the COVID-19 outbreak in China. Science. 2020;368:1481–6. 10.1126/science.abb8001 32350060 PMC7199529

[9] Principi N, Esposito S, Marchisio P, Gasparini R, Crovari P. Socioeconomic impact of influenza on healthy children and their families. Pediatr Infect Dis J. 2003;22(Suppl):S207–10. 10.1097/01.inf.0000092188.48726.e4 14551476

[10] Ferguson NM, Cummings DA, Fraser C, Cajka JC, Cooley PC, Burke DS. Strategies for mitigating an influenza pandemic. Nature. 2006;442:448–52. 10.1038/nature04795 16642006 PMC7095311

[11] Liu J, Liao X, Qian S, Yuan J, Wang F, Liu Y, et al. Community transmission of severe acute respiratory syndrome coronavirus 2, Shenzhen, China, 2020. Emerg Infect Dis. 2020;26:1320–3. 10.3201/eid2606.200239 32125269 PMC7258448

[12] Choe YJ, Smit MA, Mermel LA. Comparison of common respiratory virus peak incidence among varying age groups in Rhode Island, 2012–2016. JAMA Netw Open. 2020;3:e207041. 10.1001/jamanetworkopen.2020.7041 32401314 PMC7221508

